# A Calibration Method for the Resolution of 2D TPP Laser Direct Writing

**DOI:** 10.3390/mi14010212

**Published:** 2023-01-14

**Authors:** Yu Xie, Yixiong Chen, Hang Xu, Jianxiong Chen

**Affiliations:** School of Mechanical Engineering and Automation, Fuzhou University, Fuzhou 350108, China

**Keywords:** 2D two-photon polymerization, laser direct writing, resolution calibration, high aspect ratio voxel, 45° scanning method

## Abstract

To improve the fabrication efficiency of the two-photon polymerization (TPP) laser direct writing, the TPP exposure process was set to complete by a single-line scan, which was named 2D TPP. The voxel of the 2D TPP should be large enough to cross the photoresist and the underlayer. To explore the resolution limit of the 2D TPP considering the thickness of the photoresist, a new method named the 45° scanning method was proposed. Meanwhile, a two-photon micro-nano fabrication platform was developed. A group of experiments based on the orthogonal decomposition method was carried out to analyze the width and length of the voxel on the S1805 photoresist under different laser power and processing speed. To confirm whether the resolution of the micro-nano structures fabricated by 2D TPP is consistent with the width of the voxel, aluminum wire grids were fabricated through the 2D TPP and the metal lift-off process. A second-order regression equation of the machining resolution and input parameters of the 2D TPP is deduced. The correlation coefficient between the width of the voxel and the aluminum wire grids is 0.961, which means a significant positive correlation between them. Finally, the second-order regression model derived from the fabrication resolution of the 2D TPP was validated by experiments. Full 2D grids were fabricated using 2D TPP and mental lift-off process. This paper provides a convenient, low-cost, and high-efficiency method for calibrating the fabrication resolution of 2D TPP on various photoresists.

## 1. Introduction

Femtosecond laser direct writing has the capability of super-resolution processing. Its maskless and efficient features make it the first choice for the fabrication of micro- and nanostructures. However, the resolution of the femtosecond laser direct writing process is limited by the diffraction limit, which is positively correlated with the wavelength of the laser and negatively correlated with the numerical aperture (NA) of the objective lens. To improve the resolution of the femtosecond laser direct writing process, the optimization methods generally used are to reduce the laser wavelength or to increase the NA of the objective lens. In laser direct writing, near-infrared light at 800 nm is usually experimented with to initiate polymerization [1,2]. Reducing the laser wavelength is very costly. Meanwhile, the price of the objective lens exponentially increases with increasing NA. Both of these optimization methods are too costly. A more economical approach is to introduce the two-photon polymerization (TPP) process, which is an effective means of improving resolution [3,4]. TPP can break the diffraction limit to obtain sub-micron structures [5].

**Figure 1:**
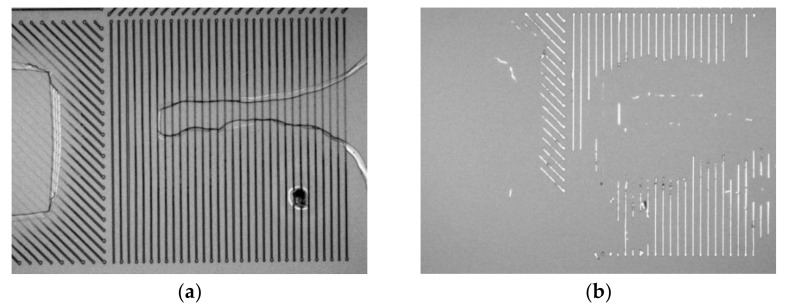
(**a**) Photoresist gratings after development, (**b**) aluminum gratings after mental lift-off process.

A typical process of TPP laser direct writing technology to fabricate micro-nano devices is as follows: (1) spin-coating the underlayer and the photoresist on the substrate, (2) exposing the photoresist by TPP laser direct writing technology, (3) developing the exposed photoresist, (4) metal deposition, and (5) lift-off process to remove the substrate [6]. To improve the manufacturing efficiency of the TPP laser direct writing process, the TPP exposure process was completed by a single line scan, which was named 2D TPP. The exposure rate in the depth direction determines the success or failure of laser direct writing. The spin-coated photoresist is generally hundreds of nanometers. In practice, we found a risk of insufficient exposure when the scanning speed is too fast under specific laser power. If the exposure dose of the photoresist is inadequate, the voxels cannot cover the vertical section of the photoresist and the underlayer. In the cationic curing system, exposure at low doses only generates a latent image of photoacids in the exposed regions (Figure 1a). The aluminum wire grids were removed along with the photoresist and the underlayer, and nothing was left (Figure 1b). To avoid failure, the laser power should increase. However, the machining width increases with the laser power. To determine the highest resolution of the 2D TPP on a specific photoresist, the present paper proposes a method to calibrate the TPP processing resolution under a single scan.

The voxel is the unit of TPP and determines the resolution of the TPP machining [7]. A theoretical equation can be used to analyze the voxel when the exposure dose of the TPP just exceeds the threshold. However, the equation does not work when the light intensity of TPP exceeds the diffraction limit [8]. The minimum spin coating thickness of commercial photoresist plus the underlayer is not less than 600nm. To achieve a single-line scan processing, the length of the voxel must be greater than 600 nm. Meanwhile, the light intensity of the TPP must be well above the threshold to ensure sufficient exposure. Empirical equations are not applicable above the threshold because of the complex photochemical reactions in overexposure situations. To analyze the machining resolution of 2D TPP, it is crucial to study the large aspect ratio voxel in overexposure situations.

Liao et al. [9] used large aspect ratio voxels under overexposure conditions for rapid fabrication. This method was used by the author to process structures with aspect ratios as high as 17. However, he did not study the relationship between processing speed and voxel. Two scans are simultaneously performed to ensure the processing success rate, so processing stability cannot be guaranteed. Devoe et al. [10] found a strong correlation between the aspect ratio of voxel and the exposure dose. The voxel aspect ratio is about one around the threshold and increases to 8–10 at high exposures. This finding is consistent with Hong-Bo Sun’s [11] results. The observation of isolated voxels is difficult. Researchers have tried a variety of methods, from single points or rods [11,12,13,14] to dangling rods [15] or lines [16]. However, a calibration method was not derived for the size of large aspect ratio voxels. In this paper, a fast calibration method for the resolution of 2D TPP was proposed to deal with situations where the Gaussian model is not applicable.

In our work, the fabrication resolution of the 2D TPP was calibrated using a new method called the 45° scanning method, and the calibration results were validated by a group of experiments. First, the principle of 2D TPP with femtosecond lasers is presented. Then, a two-photon micro-nano fabrication platform was developed to calibrate the fabrication resolution of 2D TPP by the 45° scanning method. A group of experiments was carried out based on the orthogonal decomposition method to analyze the width of the voxel on the S1805 photoresist under different laser power and processing speed. A second-order regression equation of the machining resolution and input parameters of the 2D TPP is deduced. To confirm whether the resolution of the micro-nano structures fabricated by 2D TPP is consistent with the width of the voxel, aluminum wire grids were fabricated through the combination of 2D TPP and the metal lift-off process. The correlation coefficients between the width of the voxel and the aluminum wire grids were evaluated. Finally, the derived second-order regression model of the fabrication resolution of the 2D TPP was validated by a series of experiments.

## 2. 2D TPP

### 2.1. Principles of 2D TPP

The principle of the TPP of the femtosecond laser is that the spot generated by the femtosecond laser is focused on the inside of the material to be processed, and the two-photon absorption effect occurs in this area [17]. Two-photon absorption is a third-order nonlinear optical process. The energy absorption rate of the TPP is proportional to the square of the incident light intensity, which can be expressed as:

(1) dWdt=8π2ωn2c2I2Im(χ(3))

where *dW/dt* is the energy absorption rate, *n* is the refractive index, *ω* is the frequency of incident light, *c* is the propagation speed of light in a vacuum, *I* is the intensity of incident light, and Im(*χ*^(3)^) is the imaginary part of the third order coefficient. TPP occurs only in the laser focus and a tiny area near it [18]. Therefore, the 3D microstructure processed by TPP has the advantages of high resolution and free model design. To improve the processing efficiency of the TPP laser direct writing process, the TPP exposure process was completed by a single line scan, which was named 2D TPP. 

### 2.2. Problems Caused by the 2D TPP

In the present study, the resolution represents the feature size of the micro-nano structures manufactured by 2D TPP. Working in the line scanning mode, the resolution of 2D TPP refers to the width of lines [7]. In TPP direct laser writing, the size and shape of the voxels determine the resolution [19]. The width of the exposed line on the photoresist is determined by the diameter (width) of the voxel. In the fabrication of the micro-nano structure (such as semiconductor devices), photoresist films with specific thicknesses are required. The spin-coated photoresist is generally hundreds of nanometers. The 2D TPP exposure process can be achieved by adjusting the aspect ratio of the voxels. To obtain voxels with lengths of hundreds of nanometers, the exposure dose of TPP must be higher than the threshold light intensity. The exposure rate in the depth direction determines the success or failure of laser direct writing photolithography. If the exposure dose of the photoresist is insufficient, the vertical length of the voxel is smaller than the sum of the thickness of the photoresist and the underlayer. In the cationic curing system, exposure at low doses only generates a latent image of photoacids in exposed regions [16]. The aluminum wire grids were removed along with the photoresist and the underlayer, and nothing was left. The voxels cannot cover the vertical section of the photoresist and the underlayer with decreased laser power. To avoid failure, the laser power should increase. However, the exposure width increases with increasing laser power. To obtain the thinnest gratings, the laser power and micromachining speed should be adjusted. It is essential to study the formation mechanism of voxels. The scaling law between voxel size and laser power as well as machining speed should be analyzed.

The essential parameters to characterize the structure’s writing resolution, line width, and feature size are laser power, exposure dose, and concentration of active species in the voxel after absorption [14]. Appropriate optimization of these parameters results in superior resolution. The voxel volume directly varies with the cube of the wavelength of the light source. This implies that the smaller the wavelength, the smaller the voxel dimensions. The developed line width and resolution were much superior for TPP fabrication compared with those of one photon exposure [16]. However, the concentration of active species around the voxel increased with exposure due to the presence of residual dynamic species. This effect produces undesirable polymerization surrounding the voxel, resulting in thick lines and adversely affecting the spatial resolution. The scanning speed also plays a vital role in defining the resolution of fabricated structures. At specific laser powers, the resolution increases with the increasing scanning speed, i.e., higher scanning speeds result in enhanced resolution. The reduction of exposure doses leads to a reduction in line width. However, high scan speeds can hinder the polymerization process and result in weaker and easily collapsible structures [20]. Therefore, the optimal selection of input laser power and scanning speed is vital for 2D TPP.

### 2.3. TPP Fabrication Platform

**Figure 2:**
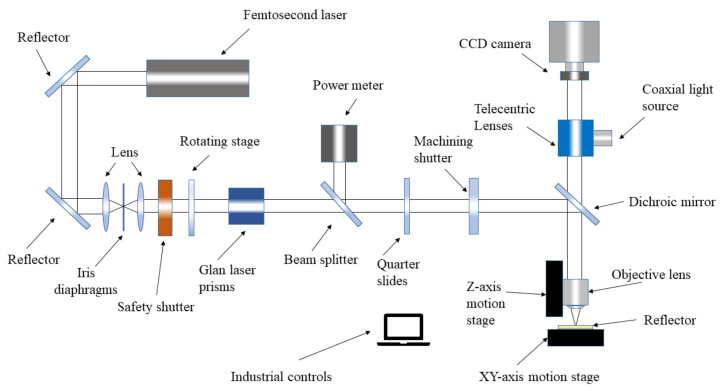
Schematic diagram of the femtosecond laser TPP micro-nano fabrication platform.

Based on the femtosecond laser TPP micro-nano processing principle, a femtosecond laser TPP micro-nano fabrication platform was established. The fabrication platform mainly consists of an optical system and a three-dimensional processing platform (as shown in Figure 2). The entire fabrication platform was built in a clean room with constant temperature, humidity, and ventilation. The Vitara Ti: sapphire femtosecond laser generator (Coherent, CA, USA) was introduced as the seed light source. The repetition frequency of the oscillator is 80 MHZ. The pulse width of the femtosecond laser is 15 fs, with an output power of 1000 mW and a center wavelength of 800 nm. Emitting from the seed light exit port of the laser generator, the femtosecond laser is reflected by a reflector and passes through a safety shutter. Then, the femtosecond laser enters the laser energy automatic adjustment system composed of a rotating stage with a half-wave plate, a Glan prism, a beam splitter, and a power meter. The processing detection software can control the rotation angle of the rotary stage to precisely adjust the processing power, and the power meter can measure the current processing power in real time. Then, the laser passes through the quarter-wave plate and enters the processing shutter. The platform uses a 650 nm short-wave pass dichroic mirror to realize the total reflection of the laser and the transmission of natural light. Finally, the laser beam is closely focused with an objective lens (magnification of 50, NA of 0.95) on photoresists. 

The three-dimensional processing platform consists of a horizontal *X*-*Y* stage and a vertical *Z* stage. A microporous ceramic vacuum adsorption platform is fixed on the *X*-*Y* stage, and the workpiece is placed on the workpiece adsorption platform during processing. The vacuum suction force generated by the adsorption platform acts to fix the workpiece on it. A lens holder is fixed on the Z-axis stage. Upon being fixed on the lens holder, the objective lens can be replaced according to different processing requirements. The relative position of the laser focus and the photoresist is adjusted by controlling the *X*-*Y* stage and the objective lens. The workpiece is processed with the opening and closing of the machining shutter.

## 3. Results and Discussion

### 3.1. 45° Scanning Method to Study Large Aspect Ratio Voxels 

**Figure 3:**
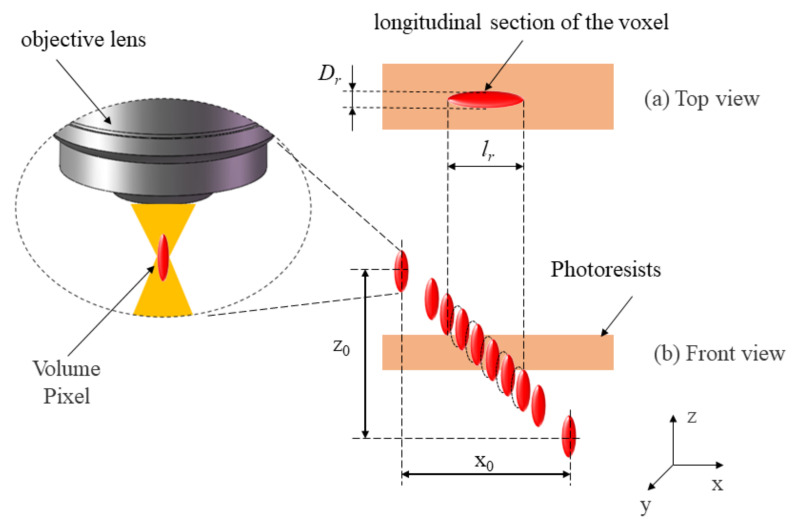
Schematic diagram of the movement of the voxel relative to the photoresist.

The following experiments were performed to study the size and shape of voxels. According to Zhou’s study [7], the laser power, the micro-machining speed, and the properties of the photoresist material are three critical parameters that influence the width ( *D_voxel_*) and length (*l_voxel_*) of the voxel. To calibrate the resolution of 2D TPP, a series of experiments based on the orthogonal decomposition method was designed. The variation area of the laser power and the machining speed is shown in Table 1, where the machining speed *V* is the combined speed of the *X* and *Z* axes speed. During the experiments, the *X* and *Z* axes of the 3D processing platform were controlled to move at the same speed (*v_x_ = v_z_*). As the TPP platform scans the photoresist at 45°, the calibration method was named the 45° scanning method. First, the laser focus was moved to the top of the photoresist. With the movement of the *X* and *Z* axes, voxels are formed on the photoresist until the laser focus leaves the photoresist (as shown in Figure 3). During this process, voxels are formed at the maximum light intensity of the laser focus, and the exposed area of the photoresist reacts. The thickness of the photoresist (*l_photoresist_*) is 500 nm. After the development of the photoresist, the reaction area was observed through the microscope, which is the longitudinal section of the voxel. The length of the reaction area *l_r_* = *l_voxel_* + *l_photoresist_*. The width of the reaction area *D_r_* = *D_voxel_*. 

**Figure 4:**
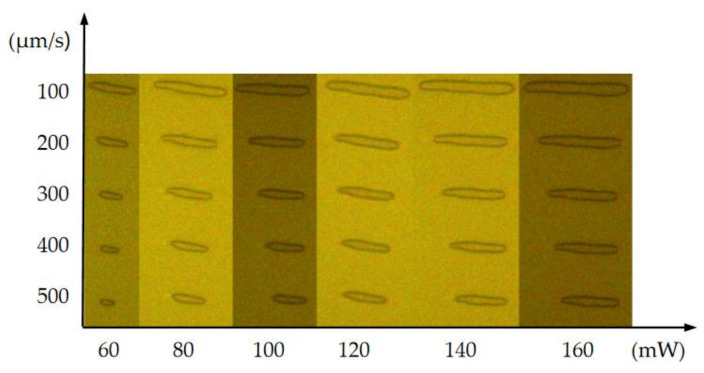
The longitudinal section of the S1805 voxel under different laser powers and processing speeds (50×).

Thirty sets of experiments were carried out on the basis of the orthogonal decomposition method. The longitudinal cross-section of S1805 voxels under different laser powers and machining speeds is shown in Figure 4. The size of the voxel increases with increasing laser power and decreases with increased processing speed. 

**Figure 5:**
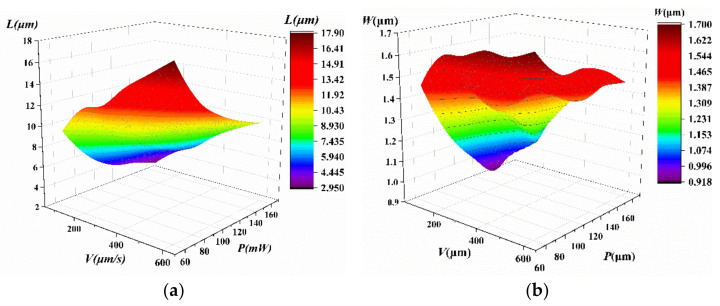
3D plots of (**a**) the length and (**b**) the width of voxels on the S1805 photoresist with respect to the laser power and the machining speed.

The width and length of the voxels in the S1805 photoresist are shown in Table 2. Figure 5a is the 3D plot of the voxel length with respect to the laser power and the machining speed. The voxel length is directly proportional to the laser power and inversely proportional to the processing speed. Figure 5b shows the 3D plot of the voxel width with respect to the laser power and machining speed. The voxel width is directly proportional to the laser power and inversely proportional to the machining speed. 

Based on Table 2, a regression analysis was performed to explore the scaling laws of *D_voxel_* and *l_voxel_* with respect to the laser power and the machining speed. A second-order regression model was introduced to study their relationship. The second-order regression model for *D_voxel_* can be expressed as follows:

(2) $$D_{\mathrm{voxel}}=1.893+0.006724P-0.003333V-2.214\times 10^{-5}P^{2}+2.774\times 10^{-6}V^{2}+7.3\times 10^{-6}PV$$

The adequacy and fitness of the model were checked by the coefficient of determination (*R*^2^). The *R*^2^ gives the proportion of the total variation in the response predicted by the model, indicating the ratio of the sum of squares due to regression to the total sum of squares [21]. The determination coefficient *R*^2^ = 0.9326, which indicates that 93.26% of the experimental data were in accordance with the values predicted by the model. 

The second-order regression model for *l_voxel_* can be expressed as follows:

(3) $$l_{voxel}=7.058+0.1147P-0.04111V-0.0001489P^{2}+4.467\times 10^{-5}PV-2.7\times 10^{-5}V^{2}$$

The determination coefficient *R*^2^ = 0.9918, which indicates that 99.18% of the experimental data were in accordance with the values predicted by the model. 

**Figure 6:**
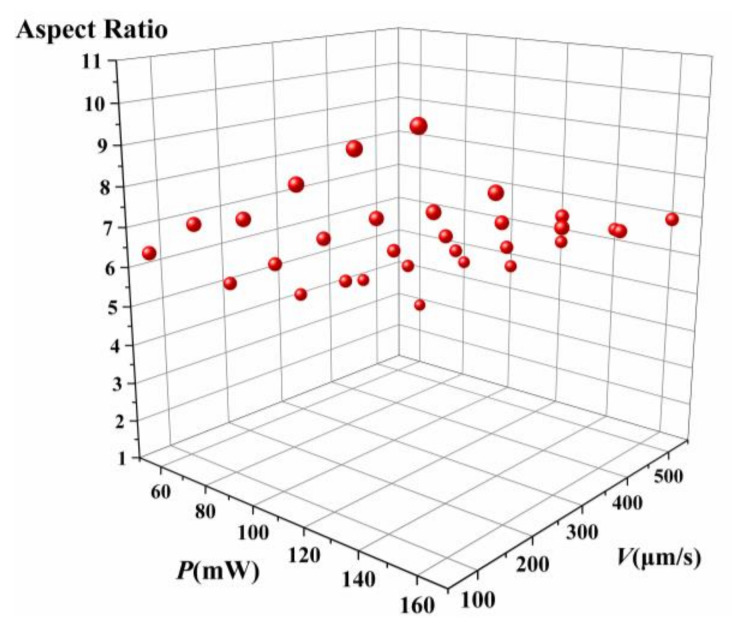
Aspect ratio of the voxel on the S1805 photoresist with respect to velocity and laser power.

Then, another critical parameter of the voxel, the aspect ratio—which is the ratio of the voxel’s length and width—was studied. For the 2D TPP, the voxel length should be as large as possible, and the voxel width should be as small as possible. Therefore, the aspect ratio of the voxel should be large enough to ensure processing accuracy and avoid insufficient exposure or exposure failure. A reasonable combination of processing speed and laser power can meet the requirements. The scatter diagram of the aspect ratio of the voxel with respect to laser power and machining speed is shown in Figure 6. The aspect ratio can reach 8.13 with a power of 160mw and a machining velocity of 100 μm/s.

### 3.2. Validation Experiments

**Figure 7:**
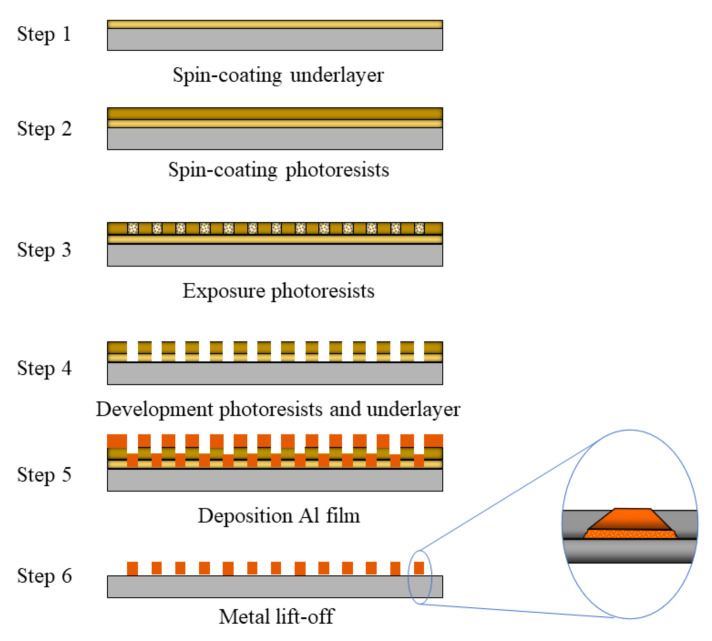
The fabrication process of micro-nano structures through 2D TPP and metal lift-off technology.

To confirm whether the resolution of the micro-nano-structures fabricated by 2D TPP is consistent with the width of the voxel, a group of micro-nano-structures was fabricated. A typical process of the TPP laser direct writing process to fabricate micro-nano structures is as follows (Figure 7): (1) the substrate (soda-lime glass) is cleaned using an ultrasonic machine with acetone, ethyl alcohol, and deionized water for 6 min each. (2) A underlayer (LOR 10B) with a thickness of 1 μm is spin-coated on the surface of the substrate. Then, the underlayer film is baked for 3 min at 150 °C (Figure 7 step 1). The underlayer is non-photosensitive but can dissolve in the ZX-238 solvent. (3) A photoresist film (S1805) with a thickness of 500 nm is spin-coated on the LOR 10B film. Then, the S1805 film is baked for 1 min at 115 °C (Figure 7 step 2). (4) 2D TPP is performed on the photoresist film, which is photopolymerizable (Figure 7 step 3). (5) The exposed photoresist film is developed by the ZX-238 solvent, and then a photoresist mask plate is obtained (Figure 7 step 4). (6) Metal lift-off technology [6] is performed. The substrate is aluminized and stripped to obtain aluminum wire grids (Figure 7 steps 5 and 6). The metal lift-off technology is a process of applying adhesive, exposing, and developing a mask with a specific shape on the substrate. After exposure and development, the substrate is aluminized and stripped to obtain aluminum wire grids. This section investigates the width of metal wire grids after aluminizing and stripping.

**Figure 8:**
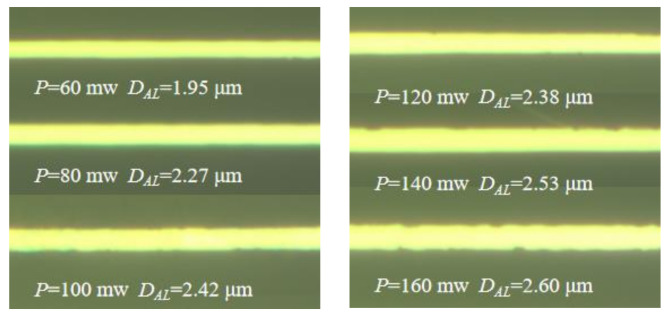
Optical microscope images of aluminum wire grids (machining speed V = 100 μm/s).

**Figure 9:**
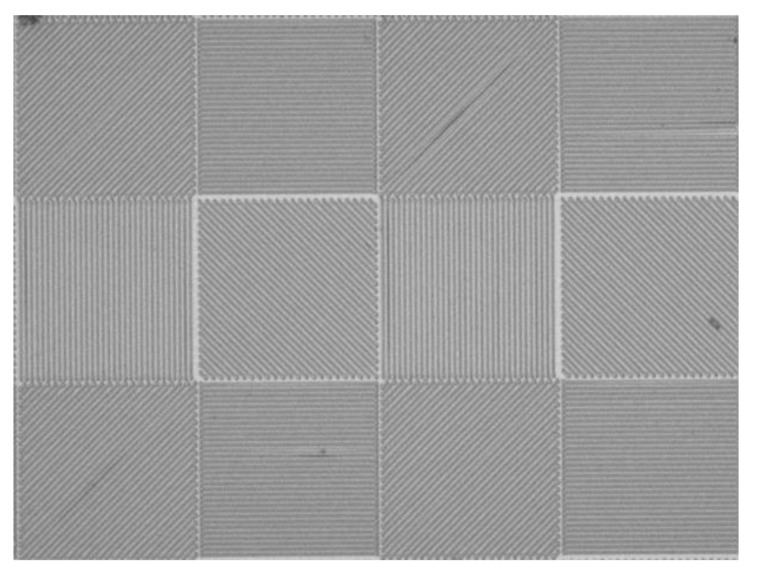
An optical microscope image of the fabricated full 2D grids (10×).

The S1805 photoresist is exposed by the femtosecond laser TPP micro-nano fabrication platform built in Section 2. The most significant factor that influences the width of the exposed line is the degree of exposure [22]. Meanwhile, the laser power and the processing speed are critical parameters that affect the degree of exposure. The S1805 photoresist was exposed to different laser powers and different processing speeds. The width of the aluminum wire grid (*D_AL_*) was measured under the microscope. As shown in Table 3, the width of the aluminum wire grid increases with increasing laser power and decreases with increased processing speed. Aluminum wire grids under a processing speed of 100 μm/s and laser power from 60 to 160 mW are shown in Figure 8. Aluminum wire grids are straight and smooth, which can meet the requirements of micro-nano structures. Full 2D grids have been fabricated using 2D TPP and the metal lift-off process. Figure 9 shows the top view of optical microscope images of the aluminum wire grids exposed with a laser power of 43 mW and a scan speed of 200 μm/s.

Based on Equation (2), the theoretical value of the width of the voxel (*D^′^*_voxel_) was obtained (Table 3). The width of the voxel is compared with the width of the aluminum wire grids. As shown in Table 3, there is little difference between *D^′^_voxel_* and *D_AL_*. Then, the covariance and the correlation coefficient of *D^′^_voxel_* and *D_AL_* were introduced to evaluate their correlation.

The covariance of *D^′^_voxel_* and *D_AL_* is defined as follows:

(4) $$Cov(D\prime_{voxel},D_{AL})=E[D\prime_{voxel}D_{AL}]-E[D\prime_{voxel}]E[D_{AL}]$$

The correlation coefficient of *D^′^_voxel_* and *D_AL_* is defined as:

(5) $$r(D\prime_{voxel},D_{AL})=\frac{Cov(D\prime_{voxel},D_{AL})}{\sqrt{Var[D\prime_{voxel}]Var[D_{AL}]}}$$

where *E*[*D^′^_voxel_*] is the expected value of *D^′^_voxel_*, *Cov*(*D^′^_voxel_*, *D_AL_*) is the covariance of *D^′^_voxel_* and *D_AL_*, *Var*[*D^′^_voxel_*] is the variance of *D^′^_voxel_*, and *Var*[*D_AL_*] is the variance of *D_AL_*. According to Eq. (4) and Eq. (5), *Cov*(*D^′^_voxel_, D_AL_*) and *r*(*D^′^_voxel_, D_AL_*) are equal to 0.048 and 0.961, respectively. For *r*(*D^′^_voxel_, D_AL_*) > 0.8, it can be derived that there is a significant positive correlation between *D^′^_voxel_* and *D_AL_*. The theoretical results calculated by Equation (2) are consistent with the experimental results. It is confirmed that the resolution of the micro-nano structures fabricated by 2D TPP is determined by the width of the voxel. The derived second-order regression model (Equation (2)) is correct.

To achieve a single-line scan processing, the length of the voxel must be greater than the thickness of the photoresist. According to the length of the voxel, the minimum width of the voxel can be calculated on the basis of Equations (2) and (3). Finally, the resolution of the 2D TPP (under a specific thickness of the photoresist) was calibrated. Our calibration method for 2D TPP works well and can be extended to other photoresists. In this section, micro-nano metal wire grids were fabricated by the combination of 2D TPP and metal lift-off technology. As for 2D TPP, other manufacturing methods (such as etching) can also be combined to fabricate micro-nano structures. The present calibration method can be extended to these processes.

## 4. Conclusions

To improve the fabrication efficiency of the TPP laser direct writing, the present paper proposed a fabrication method of completing the TPP exposure process by a single scan, which was named 2D TPP. A new calibration method, named the 45° scanning method, was designed to explore the resolution limit of the 2D TPP, considering the thickness of the photoresist. As the voxel is the unit of the TPP and determines the machining resolution of the TPP, the width and length of the voxel were investigated by a self-developed TPP micro-nano fabrication platform. The second-order regression equation of the machining resolution and input parameters of the 2D TPP was expressed as *D_voxel_* = 1.893 + 0.006724*P* − 0.003332*V* − 2.214 × 10^−5^*P*^2^ + 2.774 × 10^−6^*V*^2^ + 7.3 × 10^−6^*PV*. Combined with the metal lift-off process, 2D TPP provides a highly efficient way for the fabrication of micro-nano structures. To validate the calibration results, aluminum wire grids were fabricated through the 2D TPP and the metal lift-off process. The covariance and correlation coefficient between the width of the voxel and the aluminum wire grids are 0.048 and 0.961, respectively, which means a significant positive correlation between them. Finally, the derived second-order regression model of the fabrication resolution of the 2D TPP was validated by experiments. This paper provides a convenient, low-cost, and high-efficiency method to calibrate the manufacturing resolution of the 2D TPP. The present 45° scanning method was suitable for fast calibration of the resolution of 2D TPP on various photoresists.

## Figures and Tables

**Table 1:** Variation area of the laser power and the micromachining speed.

Num	1	2	3	4	5	6
laser power *P* (mW)	60	80	100	120	140	160
Processing speed *V* (μm/s)	100	200	300	400	500	

**Table 2:** The width (*D_voxel_*) and length (*l_voxel_*) of the voxels in the S1805 photoresist (in um).

	***P* (mW)**	60		80		100		120		140		160	
***V* (µm/s)**		*l_voxel_*	*D_voxel_*	*l_voxel_*	*D_voxel_*	*l_voxel_*	*D_voxel_*	*l_voxel_*	*D_voxel_*	*l_voxel_*	*D_voxel_*	*l_voxel_*	*D_voxel_*
100		9.00	1.96	11.56	2.11	12.35	2.13	14.37	2.17	15.88	2.16	17.39	2.2
200		5.68	1.69	7.97	1.91	9.08	1.88	10.51	1.93	11.99	2.04	13.39	2.08
300		3.94	1.53	6.16	1.83	7.47	1.8	9.14	1.92	10.31	1.96	11.44	2.08
400		3.32	1.42	5.21	1.67	6.12	1.68	7.84	1.89	9.28	1.88	10.27	2.04
500		2.47	1.56	4.22	1.56	5.35	1.76	7.12	1.85	8.4	1.92	9.44	1.96

**Table 3:** The theoretical value and the experimental value of the width of the aluminum wire grids (in µm).

	***P* (mW)V**	60		80		100		120		140		160	
***V* (µm/s)**		*D^′^*_voxel_	*D_AL_*	*D^′^*_voxel_	*D_AL_*	*D^′^*_voxel_	*D_AL_*	*D^′^*_voxel_	*D_AL_*	*D^′^*_voxel_	*D_AL_*	*D^′^*_voxel_	*D_AL_*
100		1.95	1.95	2.04	2.27	2.11	2.42	2.16	2.38	2.20	2.53	2.21	2.60
300		1.60	1.73	1.71	1.82	1.81	1.91	1.89	2.02	1.96	2.13	2.00	2.26

## Data Availability

Not applicable.
